# Pseudo Subarachnoid Hemorrhage Sign in Bacterial Meningitis in a Patient Presenting With Acute Ischemic Stroke: A Novel Radiological Clue to Rapid Diagnosis

**DOI:** 10.7759/cureus.25283

**Published:** 2022-05-24

**Authors:** Morgan E Fretwell, Naresh Mullaguri, Sanjeev Sivakumar, Mike Knipfing

**Affiliations:** 1 Neurology, Prisma Health Greenville Memorial Hospital, Greenville, USA; 2 Neurocritical Care, Prisma Health Greenville Memorial Hospital, Greenville, USA; 3 Neuroradiology, Prisma Health Greenville Memorial Hospital, Greenville, USA

**Keywords:** ischemic stroke, computerized tomography, iodine contrast extravasation, pseudo subarachnoid hemorrhage, acute bacterial meningitis

## Abstract

Pseudo subarachnoid hemorrhage (SAH) is an entity defined when characteristic computed tomography (CT) findings of SAH are seen without evidence of hemorrhage on MRI, autopsy, or cerebrospinal fluid analysis. This imaging phenomenon has been reported in association with multiple clinical settings including diffuse cerebral edema, hypoxic-ischemic injury, post percutaneous coronary intervention, and the focus of our report, acute bacterial meningitis. The mechanisms leading to this finding are poorly understood. Current hypotheses explaining this pattern vary widely depending on the associated pathology. In this report, we present a case of pseudo SAH associated with bacterial meningitis and a literature review on the causes, neuroimaging findings, and mechanisms associated with pseudo SAH. We discuss dual energy CT as a possible tool for differentiating pseudo SAH from true SAH. We analyze the timing of imaging studies and the role timing plays in the presentation of the pseudo SAH sign. We conclude that the extravasation of iodine contrast into the subarachnoid space can mimic SAH on CT. Ultimately, our case adds to the growing body of evidence that clinicians should be aware of acute bacterial meningitis as a potential mimic of SAH on CT.

## Introduction

Increased attenuation of the subarachnoid space on computed tomography (CT) of the head is a characteristic finding of acute subarachnoid hemorrhage (SAH). However, several radiographic mimics of SAH have been reported [1-2]. Pseudo subarachnoid hemorrhage (pSAH) is an entity defined when characteristic CT findings of SAH are seen without evidence of hemorrhage on autopsy or cerebrospinal fluid (CSF) analysis. The most common causes of pSAH are diffuse cerebral edema, hypoxic-ischemic injury, post percutaneous coronary intervention, and acute bacterial meningitis (ABM) [1]. Prompt diagnosis of ABM can be difficult due to its nonspecific presentation. This diagnosis is further confounded in nearly 20% of patients who present with stroke-like symptoms [3-4]. Delay in diagnosis and management of ABM can significantly increase mortality by 12.6% per hour of delay [5]. In this report, we discuss a patient with ABM initially presenting with symptoms of stroke and with neuroimaging findings of the pSAH sign, who suffered a poor clinical outcome due to delay in diagnosis and treatment. In addition, we provide a literature review on cases of pSAH that were attributed to an infectious etiology, their clinical and radiographic characteristics to highlight that pSAH sign is a potential clue for the early diagnosis of ABM.

## Case presentation

**Figure 1:**
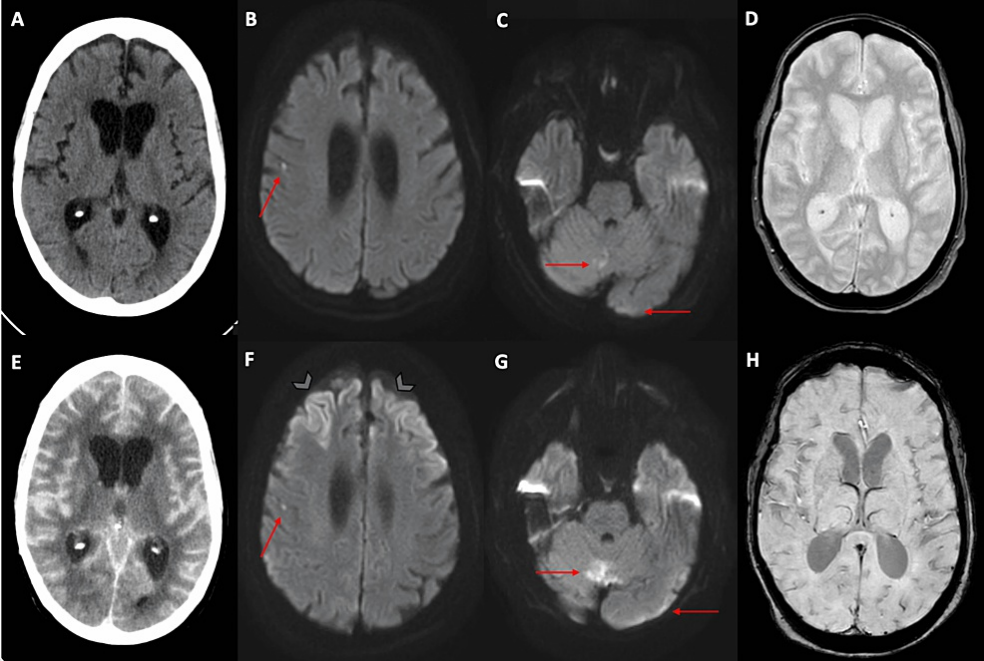
Initial and repeat imaging findings (A) Initial CT (axial section) of the head with contrast showing no subarachnoid hemorrhage. (B, C) Initial MRI of the brain (axial section, diffusion-weighted imaging) showing acute punctate infarct in the right frontal lobe and cerebellum (red arrows). (D) Initial MRI of the brain (susceptibility-weighted imaging) without signs of bleeding in the subarachnoid space. (E) Repeat CT (axial section) of the head with contrast after six hours showing hyperattenuation in subarachnoid space concerning SAH. (F, G) Repeat MRI of the brain (axial section, diffusion-weighted imaging) after six hours showing hyperintensity of bilateral frontal lobes (blue arrowheads) as well as punctate intensities in the right frontal lobe and cerebellum (red arrows). (H) Repeat MRI of the brain (susceptibility-weighted imaging) after six hours showing no dark signal in subarachnoid space to indicate true SAH.

A 64-year-old Caucasian female with alcohol abuse and chronic thrombocytopenia was brought to the emergency department, after being found with confusion, aphasia, and right-sided weakness. On arrival, she was alert with a chief complaint of severe headache. Her initial National Institutes of Health Stroke Scale was 23 for a diminished level of consciousness, aphasia, and motor deficits. Specifically, she received points for arousal to minor stimulation, inability to answer questions or follow commands, bilateral upper extremity drift, no effort against gravity in the left leg, no movement in the right leg, mild paresthesias, severe aphasia and dysarthria, and profound hemi-inattention. The CT scan of the head (Figure 1A) was unremarkable for any hemorrhage or early ischemic changes. A CT angiogram of the head and neck revealed no evidence of hemodynamically significant stenosis or saccular aneurysm. At this point, it was felt that the patient may have had a seizure as well as an ischemic stroke and acute metabolic encephalopathy. The patient was not considered a candidate for tissue plasminogen activator (tPA) due to the unknown time of symptom onset. MRI of the brain showed small acute infarcts present within the right frontal lobe (Figure 1B), superior paramedian right cerebellar hemisphere, and left occipital lobes (Figure 1C). Susceptibility-weighted imaging revealed no signs of bleeding in the subarachnoid space (Figure 1D). Six hours after initial presentation, the patient had an acute change in mentation and became unresponsive with bilateral fixed pupils. Repeat CT scan of the head at this time showed increased attenuation throughout the supratentorial and infratentorial subarachnoid spaces concerning SAH (Figure 1E). The patient was admitted to the neurology intensive care unit and received a loading dose of intravenous (IV) valproic acid as well as IV dexamethasone. Eight hours after admission, a repeat MRI of the brain revealed progressive diffusion restriction involving the frontal lobes (Figure 1F), and progressive foci of diffusion restriction involving the cerebellum bilaterally (Figure 1G). There was diffuse fluid-attenuated inversion recovery (FLAIR) non-suppression in the sulci to correspond to the diffuse sulcal hyperattenuation on the preceding CT; however, there was no corresponding sulcal susceptibility signal to suggest blood products (Figure 1H). Given the lack of susceptibility signal on MRI, it became evident that the preceding diffuse sulcal hyperattenuation on CT was due to extensive subarachnoid leakage of iodinated contrast administered during the CT angiogram. Differential considerations included hypoxic-ischemic encephalopathy, cerebral vasoconstriction, as well as infectious or inflammatory encephalitis.

**Figure 2:**
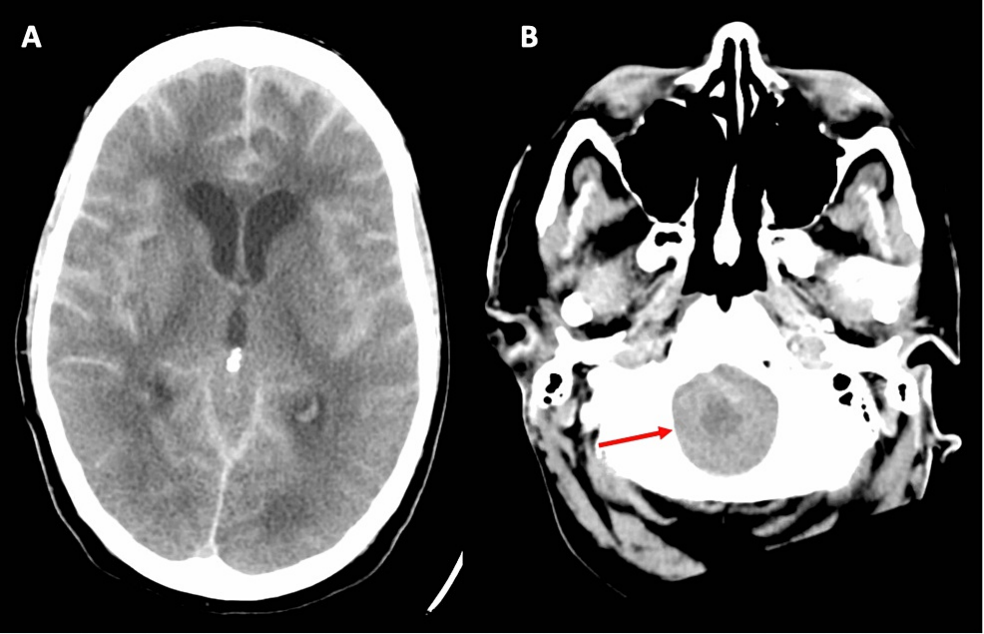
Final CT findings (A) Repeat CT (axial section) of the head with contrast after 15 hours showing decreased gray-white matter differentiation and fading intensity of the subarachnoid hyperintensity. (B) Repeat CT (axial section) of the head with contrast after 15 hours showing cerebellar tonsillar herniation through foramen magnum (red arrow).

After follow-up imaging, the patient became hypotensive and hypoxic, requiring increased vasopressor support and emergent intubation. Repeat labs were significant for a procalcitonin of 122. The patient remained afebrile and in refractory septic shock. Repeat CT of the head 15 hours after initial presentation revealed decreased gray-white matter differentiation (Figure 2A) as well as herniation of the cerebellar tonsils through the foramen magnum (Figure 2B). These findings were concerning for global cerebral edema. Of note, these images showed decreased hyperattenuation of contrast within the subarachnoid spaces when compared with imaging from nine hours prior.

At this time, CSF obtained via lumbar puncture revealed 1515 white blood cells, 2000 red blood cells, 1556 total nucleated cells, glucose of 2, and protein of 1028. These results were consistent with ABM. Empiric antibiotic therapy was started with vancomycin, cefepime, and ampicillin. Despite aggressive management, she continued to be in refractory septic shock with multiorgan failure. Given the poor prognosis, family members transitioned her to comfort measures; the patient was compassionately extubated and deceased. Blood culture and CSF culture ultimately grew beta-hemolytic group A streptococci.

## Discussion

The appearance of SAH on CT associated with ABM is reported infrequently in the medical literature. Our literature review in PubMed until January 2022 for reported cases of the pSAH sign identified 11 cases attributed to infectious etiology of which two were excluded. Two cases were excluded as full manuscripts were unavailable. Nearly half of reported cases presented with focal neurological deficits contributing to the presumption of true SAH. The remaining half presented with headache and/or encephalopathy. More often than not, head CT was obtained less than 24 hours after admission. Specific imaging patterns were roughly split between diffuse hyperdensity and focal hyperdensities found within the subarachnoid spaces. Imaging not infrequently showed increased density diffusely within the basal cisterns and along the Sylvian fissures. Nearly all of the cases resulted in death or residual sensory deficits, with only two cases showing complete resolution (Table 1) [6-14]. We present a case of a patient who presented with headache and focal neurologic deficits and had imaging findings consistent with diffuse SAH. In fact, this patient had underlying ABM with imaging findings likely representing contrast in the subarachnoid space appearing similarly to blood.

**Table 1:** List of published cases of the pSAH sign attributed to infectious etiology: clinical characteristics, neuroimaging findings, lab findings, and outcome AMS: altered mental status; CHF: congestive heart failure; CKD: chronic kidney disease; CSF: cerebrospinal fluid; CT: computed tomography; DM: diabetes mellitus; HA: headache; HIV/AIDS: human immunodeficiency virus/acquired immunodeficiency syndrome; HTN: hypertension; RBC: red blood cell; SAH: subarachnoid hemorrhage; WBC: white blood cell

Publication	Age/sex	Medical comorbidities	Presenting symptoms	Neurological symptoms	Interval between presentation and imaging	CT findings	CSF	Outcome
Chatterjee et al., 2003 [6]	43/F	History of venous thrombosis, severe eczema	Flu-like symptoms	Decreased consciousness	-	Non-contrast CT scan showing increased density within the basal cisterns and along the Sylvian fissures bilaterally	Opening pressure >35 cmH_2_O, WBC 1510×10^6/L, RBC 160×10^6/L, protein 2291 mg/L. Positive for pneumococcal antigen	Discharged; residual bilateral blindness
Given et al., 2003 [7]	6/M	-	-	-	-	Compression and/or mass effect on the fourth ventricle, effacement of the basal cisterns and cortical sulci, decreased grey-white matter differentiation	-	Death
Cucchiara et al., 2004 [8]	22/M	-	Fever, coma, seizures	Coma, seizures	-	Diffuse sulcal effacement, obliterated basal cisterns, and a dense linear area in the interhemispheric fissure	Leukocytes 44/mm^3^, with lymphocytic predominance; erythrocytes 3/mm^3^, without xanthochromia; normal protein and glucose; negative Gram stain	Resolution
Hoque et al., 2008 [9]	50/M	-	HA, confusion, vision loss	Vision loss	1 hour	CT with and without contrast showed subarachnoid hemorrhage with associated cerebral infarction in the right parietal area	Clear colorless fluid, opening pressure >35 cmH_2_O, normal glucose, no RBCs. Protein 102 mg/dL. WBC 169/mm^3^ 54% lymphocytes and 40% granulocytes. Cryptococci were detected	Discharged; residual visual acuity bilaterally
Coady et al., 2011 [10]	42/M	HIV/AIDS (CD4 T cell count of 35)	Falls, somnolence, occipital HA	Right lateral gaze palsy, bilateral horizontal and vertical nystagmus	-	Subarachnoid hemorrhage along the cisterns with effacement of the quadrigeminal cisterns	CSF was clear, WBC 2/mm^3^, RBC 73/mm^3^, total protein 5 mg/dL. A second tube’s WBC was 1 cell/mm^3^, and RBC 12 cells/mm^3^. CSF culture grew *Cryptococcus neoformans*	Death
Lang et al., 2013 [11]	57/M	HTN	Occipital HA, neck stiffness, confusion, vomiting	Expressive dysphagia	16 hours	Hyperdense substance in the occipital horns of the lateral ventricles and in the left Sylvian fissure associated with early hydrocephalus	CSF was yellow-green and turbid. Glucose 1.7 mmol/L, protein 8.1 g/L, WBC 2404, RBC 111. Grew pneumococcal meningitis	Discharged; residual Bell's palsy
Nakae et al., 2013 [12]	68/F	Ovarian tumor	HA, fever, neck stiffness, drowsiness	Decreased hearing, decreased vision	1 month	Iso- to high-density areas within the cortical sulci	Opening pressure 14 cmH_2_O, WBC 37/mm^3^ (32 lymphocytes/5 neutrophils), protein 38 mg/dL, glucose 21 mg/dL. Culture yielded *Cryptococcus neoformans*	Discharged; residual hearing and vision loss
Ho et al., 2018 [13]	83/M	HTN, hyperlipidemia, CHF, Atrial fibrillation on warfarin, DM, CKD on hemodialysis	AMS, emesis	Left gaze preference, left arm and leg in a tonically flexed position with intermittent rhythmic jerks	4.5 hours	Non-contrast CT revealed diffuse hyperdensity within the basal cisterns, Sylvian fissure, and cerebral sulci bilaterally, concerning diffuse SAH	Nucleated cell count 683 cells/μL, RBC 2180 cells/μL, glucose 69 mg/dL, protein 590 mg/dL, and Gram stain with rare Gram-positive cocci	Death
Camacho et al., 2019 [14]	22/M	Recent methamphetamine use	HA, nuchal rigidity	-	<24 hours	Noncontract brain CT with hyperdense material along the inferior right tentorial leaflet and right brainstem	Cloudy CSF. Opening pressure 48 mmHg. Glucose <10 mg/dL, protein 846 mg/dL, nucleated cell count 21.5×10^9/L, 96% segmented neutrophils. Gram stain grew methicillin-sensitive *Staphylococcus aureus*	Resolution

Several mechanisms have been proposed to explain the pSAH pattern. Mechanisms associated with meningitis suggest that toxins and inflammatory infiltrates in the CSF can compromise the blood-brain barrier (BBB) and allow leakage of proteinaceous exudate into the basal cisterns and subarachnoid space [2,11]. This explanation requires that protein-rich fluid appears hyperdense on CT, however, Norman et al. report that pathological elevation of CSF protein does not contribute to significantly increased absorption on CT [15]. It is helpful to look at pSAH cases attributed to noninfectious etiologies as well. Several reports associate the pSAH appearance with extravasation of contrast into the subarachnoid space following angiography [16-19]. These reports suggest that the contrast medium directly induces disruption of the BBB [17-18]. In these cases, the pSAH sign was visualized on CT around 4-12 hours from the time of contrast administration [16-19].

We take the two previously discussed mechanisms together to conclude that in our patient, leptomeningeal inflammation secondary to meningoencephalitis allowed contrast administered during initial CT to leak into the subarachnoid space. On follow-up CT, this contrast extravasation appeared similarly to blood leading to the pSAH appearance. This idea has been considered previously to explain the pSAH appearance in a patient with concurrent renal failure and acute meningitis [13]. That being said, Ho et al. attribute contrast extravasation to decreased clearance in the setting of renal failure. Conversely, we suggest that a leaky BBB secondary to leptomeningeal inflammation is sufficient to allow significant extravasation of contrast. Similar to other reports of pSAH following contrast administration our case demonstrates a delay of roughly six hours from contrast administration to pSAH appearance on CT [13,16-19]. Further investigation is needed to establish a causative relationship between severity of inflammation and speed of contrast extravasation.

## Conclusions

We hypothesize that in our case, contrast administered during the initial CT of the head leaked through dilated leptomeninges and into the subarachnoid space. This gave the appearance of SAH on follow-up CT of the head six hours later. Consideration of meningitis as a possible radiographic mimic of SAH might have resulted in both earlier diagnosis and treatment and changed this patient’s outcome. Physicians should be aware of potential false-positive SAH on head CT when treating patients with encephalopathy or focal neurologic deficits. Though the value of a single case report is limited in generalizability, additional reports of the pSAH appearance bring us closer to an understanding of the pathophysiology contributing to this finding.
